# Zeranol Down-Regulates p53 Expression in Primary Cultured Human Breast Cancer Epithelial Cells through Epigenetic Modification

**DOI:** 10.3390/ijms12031519

**Published:** 2011-02-25

**Authors:** Weiping Ye, Pingping Xu, Robert Jen, Eric Feng, Saiyi Zhong, Hong Li, Shu-Hong Lin, Jie-Yu Liu, Young C. Lin

**Affiliations:** 1 Laboratory of Reproductive and Molecular Endocrinology, College of Veterinary Medicine, The Ohio State University, Columbus, OH 43210, USA; E-Mails: ye.44@osu.edu (W.Y.); xu.200@osu.edu (P.X.); jenrt@mail.uc.edu (R.J.); fanx40@gmail.com (E.F.); zhong.66@osu.edu (S.Z.); li.801@osu.edu (H.L.); lin.833@osu.edu (S.-H.L.); liu.1048@osu.edu (J.-Y.L.); 2 333 Goss Laboratory, 1925 Coffey Road, The Ohio State University Comprehensive Cancer Center, Columbus, OH 43210, USA

**Keywords:** breast cancer, growth promoter, zeranol, p53, DNMT1

## Abstract

Epidemiological studies have suggested that there are many risk factors associated with breast cancer. Silencing tumor suppressor genes through epigenetic alterations play critical roles in breast cancer initiation, promotion and progression. As a growth promoter, Zeranol (Z) has been approved by the FDA and is widely used to enhance the growth of beef cattle in the United States. However, the safety of Z use as a growth promoter is still under debate. In order to provide more evidence to clarify this critical health issue, the current study investigated the effect of Z on the proliferation of primary cultured human normal and cancerous breast epithelial cells (PCHNBECs and PCHBCECs, respectively) isolated from the same patient using MTS assay, RT-PCR and Western blot analysis. We also conducted an investigation regarding the mechanisms that might be involved. Our results show that Z is more potent to stimulate PCHBCEC growth than PCHNBEC growth. The stimulatory effects of Z on PCHBCECs and PCHBCECs may be mediated by its down-regulating expression of the tumor suppressor gene p53 at the mRNA and protein levels. Further investigation showed that the expression of DNA methylatransferase 1 mRNA and protein levels is up-regulated by treatment with Z in PCHBCECs as compared to PCHNBECs, which suggests a role of Z in epigenetic modification involved in the regulation of p53 gene expression in PCHBCECs. Our experimental results imply the potentially adverse health effect of Z in breast cancer development. Further study is continuing in our laboratory.

## 1. Introduction

Breast cancer remains a serious problem in the U.S. It is estimated that more than one-fourth of cancer patients were breast cancer patients in 2010 and it ranks as the second leading cause of cancer-related deaths [1]. The risk factors associated with breast cancer have been well studied. Epidemiological studies suggest that there are many risk factors, such as dietary fat and environmental estrogenic endocrine disruptors. It has been reported that breast cancer cells express high levels of aromatase, which can convert androgens into estrogens, resulting in high concentrations of estrone (*E*_1_) and estradiol (*E*_2_) in breast tissue [2,3]. This finding might be the major reason for high risk of breast cancer in postmenopausal women and obese women.

Zearalenone, a stable natural product that mimics estrogen activity, is carcinogenic and hazardous to human health [4]. It was reported that zearalenone may represent a growth promoter in exposed patients and there is a possible relationship between environmental mycoestrogen exposure and the development of central precocious puberty [5]. Zeranol (Z), produced from zearalenone, is a non-estrogenic anabolic growth promoter and is widely used in the U.S. beef industry to accelerate weight gain, improve feed conversion efficacy, and increase the lean meat-to-fat ratio [6]. It is approved by the FDA based on its toxicity information. However, the potentially adverse health concerns associated with Z have caused the European Union (EU) to refuse the import of beef products with any growth promoter implantation from the U.S., resulting in a serious legal issue between the U.S. and the EU. We need greater concrete evidence to clarify this important health issue in order to bring awareness to the FDA.

Both zearalenone and Z can bind to the active site of human estrogen receptor α (ER α) and ER β in a manner similar to 17β-estradiol [7]. As a food contaminant, the intake of Z is very hard to avoid [8]. It is still not clear whether Z and its metabolites cause any adverse health risks from consuming beef products produced from Z-implanted beef cattle. Researchers have found that low concentrations of Z can increase ER α-positive human breast cell growth, but a high concentration of Z can reduce growth of both ERα-positive and ER-negative cell lines [9]. Our previous data showed that Z was able to transform normal human breast epithelial cells and increase human breast cell growth in a dose-dependent manner [10]. Z can also down-regulate the estrogen-regulated human breast cancer candidate suppressor gene, protein tyrosine phosphatase γ (PTPγ) expression [7]. Our laboratory’s recent experimental results showed that pre-adipocytes isolated from the cattle 60 days post Z-implantation (72 mg/pellets/animal) grow about 12-times faster than the pre-adipocytes isolated from control cattle [11]. Treatment with 0.2, 1 and 5% Z sera (ZS) harvested from the cattle 30 days post implantation in culture medium significantly stimulated MCF-10A and MCF-7 cell growth [12,13]. We also have evidence showing that leptin, which plays a role in breast cancer development in obesity, induces human breast cancer epithelial cell sensitivity to Z [14].

The relationship between the consumption of beef products with residue of Z and its metabolites and cancer development is still in debate. However, epidemiology studies showed that the semen quality of fertile U.S. males is related to their mother’ beef consumption during their pregnancy [15]. It has been reported that higher red meat intake in adolescence may increase the risk of premenopausal breast cancer [16]. Early in 1989, Aw and colleagues reported that workers were exposed to Z formulate pellets along with a package of animal growth promoter containing Z as its active ingredient in a small manufacturing plant. Their industrial exposure to Z caused one child to be afflicted with advanced bone aging due to his parents wearing their work clothes home (estimated 32 mg of Z on contaminated work clothes) [17]. It is well-known that the tumor suppressor gene p53 plays an important role in controlling cell life and death [18]. Loss of normal p53 function occurs in all types of human tumors, including breast cancer [19–21]. Baker and co-worker determined the p53 mutation status in 246 women with primary breast cancer and identified the p53 mutations in 26% of patients that may also be associated with poor prognosis [22]. It is estimated that at least one-third of non-familial breast cancer patients bear mutations in p53 [23]. Additionally, mutation of p53 is correlated with heritable mutations in BRCA1 [24]. The mechanisms of regulating p53 expression in breast cancer cells have been well studied. Our previous investigation found that the pre-adipocytes derived from the heifer after two month of Z-implantation grew about 12-times faster than those from the control heifer. The expression of p53 mRNA and protein was also lower in the Z treated group than the control [11]. However, the function of growth promoter-Z on regulation of p53 in primary cultured human normal and cancerous breast epithelial cells is still unknown.

Epigenetics is the study of nuclear inheritance that is not based on differences in DNA sequences, but the changes in DNA structure partially due to altered DNA methylation. DNA methyltransferase 1 (DNMT1) is known to be a major DNA methyltransferase that can catalyze a methyl group in cytosine residues within CpG dinucleotides and lead to the tumor suppressor gene silencing [25–27]. CpG methylation in the p53 promoter region was detected in breast cancer [28]. Our investigation has centered around determining whether Z stimulates breast cancer cell lines as well as primary cultured human normal and cancerous breast epithelial cells mediated by regulating DNA methyltransferase 1 (DNMT1) and hypermethylation in the promoter regions, thus resulting in the silencing of the tumor suppressor gene, p53 expression. The current study investigates the effect of Z on the growth of primary cultured human normal and cancerous breast epithelial cells as well as the underlying mechanisms. Our results indicate that Z is more potent to stimulate primary culture human breast cancer epithelial cells (PCHBCECS) growth than primary culture human normal breast epithelial cells (PCHNBECS) and the stimulatory effects may be mediated by regulating the expression of DNMT1 and down-regulating p53 expression in PCHBCECs but not in PCHNBECs.

## 2. Results and Discussion

### 2.1. Z Increases the Proliferation of PCHNBECs and PCHBCECs

**Figure 1.**
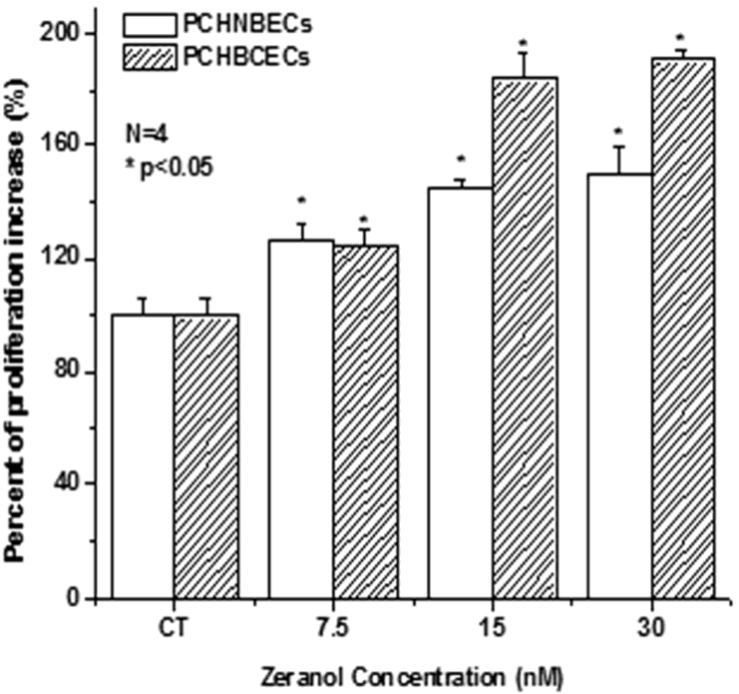
Effects of zeranol on the proliferation of PCHNBECs and PCHBCECs. PCHNBECs and PCHBCECs (5 × 10^3^ cells/well) were treated with 0.1% DMSO (as a vehicle control), 7.5, 15 and 30 nM zernaol in 96-well plates for 24 h. Non-radioactive cell proliferation assay was performed after 24 h treatment. Each bar represents the mean ± SD of 4 replicate wells. The asterisks show statistically significant differences between Z treatment groups and the control groups (*p* < 0.05). The figure illustrates that PCHBCECs are more sensitive to Z treatment than PCHNBECs.

Our previous experimental results showed that Z stimulates MCF-7 cell and MCF-10A cell growth [10]. The serum harvested from the heifer one month post Z implantation is more potent in stimulating human breast cancer cell lines as well as PCHBCEC growth than that from control heifers. In the current study, we evaluated the effects of Z on PCHNBEC’s and PCHBCEC’s growth. The results show Z increased the proliferation of both PCHNBECs and PCHBCECs in a dose-dependent manner (Figure 1). PCHBCECs are more sensitive in response to Z treatment than PCHNBECs (Figure 1).

### 2.2. Regulatory Effects of Z on p53 mRNA and Protein Expression in PCHNBECs and PCHBCECs

**Figure 2.**
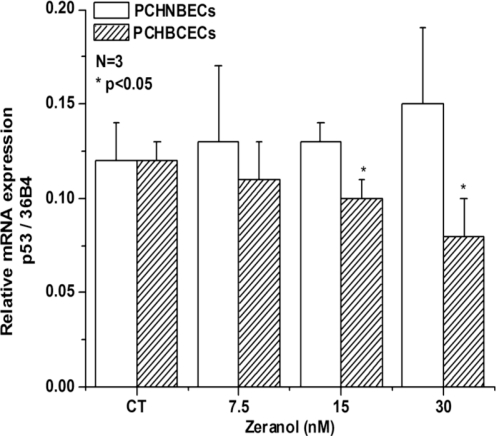
Comparison of effect of Z on the expression of p53 mRNA in PCHNBECs and PCHBCECs. PCHNBECs and PCHBCECs (1 × 10^5^ cells/well) were seeded in 6-well plates and treated with 0.1% DMSO, 7.5, 15, and 30 nM Z. After 24 h treatment, total RNA was isolated from each treatment group and RNA concentrations were measured. Then, cDNA was synthesized and real time PCR was performed to amplify p53 and 36B4 genes. The mRNA ratios of p53 to 36B4 were calculated using the ΔΔCt method. Each bar represents mean ± SE of three independent experiment. The asterisks show statistically significant differences between Z treatment groups and the control groups (*p* < 0.05). It shows that Z decreases the expression level of p53 mRNA in PCHBCECs but not in PCHNBECs.

In order to explore the possible mechanisms involved in Z stimulation of PCHNBECs and PCHBCECs proliferation, we examined the expression of the p53 gene at the mRNA and proteins level. p53 is involved in many important physiological processes such as cell cycle arrest, gene transcription, DNA repair, and apoptosis. Our result illustrated that Z treatment significantly suppressed the level of p53 mRNA and protein expression in PCHBCECs in comparison to the levels in the control group (Figures 2 and 4). However, Z did not show significant reduction of p53 mRNA and protein expression in PCHNBECs when compared to the control (Figure 2).

### 2.3. Z Regulates the Expression of DNMT1 mRNA and Protein in PCHNBECs and PCHBCECs

**Figure 3.**
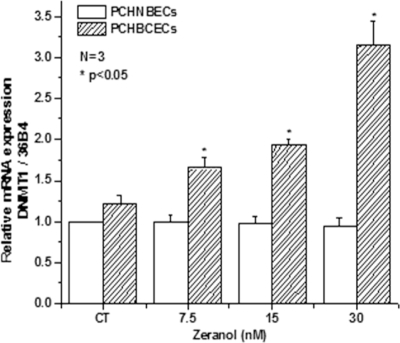
Effects of Z on regulation of the expression of DNMT1 mRNA in PCHBCECs and PCHNBECs. The expression levels of DNMT1 mRNA were evaluated in PCHBCECs and PCHNBECs after treatment with vehicle control or 7.5–30 nM Z using real time PCR analysis. 36B4 was used as an internal control. The mRNA ratios of DNMT1 to 36B4 were calculated using the ΔΔ Ct method. The asterisks show statistically significant differences between Z treatment groups and the control groups (*p* < 0.05).

**Figure 4.**
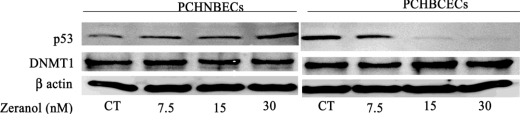
Zeranol regulates the expression of p53 and DNMT1 proteins in PCHNBECs and PCHBCECs. PCHNBECs and PCHBCECs were seeded in 10 cm culture dishes at a density of 1 × 10^6^ cells/dish and then treated with 7.5, 15, 30 nM Z or 0.1% DMSO as a vehicle control for 24 h. Proteins were extracted from each treatment group and Western blot analysis was conducted. β-actin was used to confirm equal loading of total proteins during SDS-PAGE.

DNMT1 plays an important role in silencing tumor suppressor genes, such as BRCA1, p16, and p21 in breast cancer development. It was reported that DNMT1 is over-expressed in breast cancer [29,30]. To explore the possible mechanisms of Z regulating p53 gene expression, we investigated the effects of Z on DNMT1 mRNA and protein expression in PCHNBECs and PCHBCECs after 24 h treatment. The results are shown in Figures 3 and 4. It is illustrated that Z treatment significantly increased the expression of DNMT1 mRNA and protein levels in PCHBCECs in a dose-dependent manner when compared to the control. However, Z did not significantly increase the expression of DNMT1 mRNA and protein in PCHNBECs when compared to the control. Our results suggest that Z regulate the expression of p53 in PCHBCECs, maybe through its epigenetic modification.

### 2.4. Discussion

Breast cancer development occurs in multiple stages, which are triggered by the evolution of altered gene function. Many of the gene changes that disrupt their coding regions are involved in initiation, promotion and progression [31]. P53 is a tumor suppressor gene which plays a crucial role in regulating cell growth following exposure to various stress stimuli. It induces growth arrest or programmed cell death (apoptosis) and it also plays an important role in the control of cell cycle checkpoints. Research found that p53 is the most frequently mutated gene in human cancers. An estimated 15–60% of breast cancers contain p53 mutations [32] and loss of p53 occurs in approximately 80% of colorectal tumors [33]. Consequently, its function has been extensively studied. It was reported that tumor cells with mutant p53 protein might be associated with poor prognosis [32].

The tumor suppressor p53 can regulate both cell proliferation and apoptosis. An imbalance between cell proliferation and apoptosis results in a rapid increase in cell number, the most prominent characteristic of tumors. Normal breast epithelial cells induce p53-dependent apoptosis and p53-independent cell cycle arrest of breast cancer cells [34]. In addition, p53 was also expected to play an important role in cancer treatment with its mutation predicting a substantially worsened prognosis [33]. Several retrospective studies have suggested p53 gene mutation as an adverse prognostic indicator in breast cancer patients; it can predict early recurrence, sensitivity to chemotherapy, and death in breast cancer patients [35].

Recently, it is becoming understood that epigenetic events, or heritable changes in gene expression capacity without DNA sequence alterations, are also central to tumor progression. Understanding the differential roles of regional hypermethylation and global hypomethylation in breast cancer will facilitate novel therapeutic approaches to breast cancer therapy. It was found that hypomethylation of the global genome can lead to genomic instability that is exemplified by misalignments, DNA breakage, deletions and duplications during DNA replication [36].

DNA methylation is one of the major epigenetic modifications in cancer development which has a long-standing relationship with gene inactivity and has been implicated as an important factor in gene silencing [37,38]. In humans and most mammals, DNA methylation is the only known natural modification of DNA and only affects cytosines at the 5′ position when it is followed by a guanosine, known as CpG dinucleotides. Depending on the cell types and tissue, 3–4 % of all cytosine residues in vertebrate DNA may be present as 5-methylcytosine and approximately 70 to 80% of all CpG dinucleotides in human DNA are methylated [39]. However, this methylation occurs primarily in areas where CpG density is low, or at repeat DNA sites. Most CpG islands, especially those located in promoter region of a specific gene, are completely unmethylated. Interestingly, fully methylated CpG islands are found only in the promoters of silenced alleles for selected imprinted autosomal genes and multiple silenced genes on the inactivated X-chromosomes of females [40]. It is well established that almost half of the tumor suppressor genes that cause familial cancers through germline mutations can be inactivated in association with promoter hypermethylation in sporadic cancers [41]. Huang and colleagues, using a novel DNA array based technique called differential methylation hybridization, performed a genome wide screen for hypermethylated CpG islands in a panel of breast cancer cell lines. They found that approximately 10% of CpG islands exhibited methylation in normal breast epithelial cells. In contrast, breast cancer cell lines show methylation at a considerably larger fraction of CpG islands (15–25%) [42]. In clinical research, it was reported that DNA methylation in serum of breast cancer patients may be considered as an independent prognostic marker [43]. Methylation-dependent gene silencing is now thought to be mediated through local changes in chromatin conformation that limit promoter accessibility [44].

DNA methyltransferases (DNMTs) are critical enzymes that regulate DNA methylation. DNMT1 is the most important one; the *N*-terminus of DNMT1 contains regions responsible for targeting the enzyme to replication foci [45], as well as for discriminating between unmethylated and hemi-methylated DNA [46]. Overexpression of all the DNMTs at the mRNA level has been shown for several cancers [47–49]. Conversely, a mouse model for colon cancer demonstrated that reducing DNMT1 expression and activity via hemizygous knockout of the gene and treatment with a DNMT1 inhibitor greatly reduced the number of intestinal adenomas [50]. Similarly, Macleod and Szyf found that transfection of a murine adrenocortical tumor cell line with DNMT1 antisense expression vectors resulted in general DNA hypomethylation, growth inhibition *in vitro*, and decreased tumorigenicity *in vivo* [51]. Our laboratory recently reported PTP γ expression was reduced in breast cancer cell lines, SK-Br-3 and MCF-7 cells, and it can be re-activated with the treatment of deoxy-5-azacytidine, a well-known DNMT1 inhibitor [52]. These results suggest that DNMT1 can be thought of as a new target for novel drug development.

Our data indicated that 24 h Z treatment stimulated both PCHNBEC’s and PCHBCEC’s proliferation in a dose-dependent manner, and PCHBCECs were more sensitive to Z than PCHNBECs. Z increased the DNMT1 expression in PCHBCECs and decreased the p53 expression at both the mRNA and protein levels; however, no such effect was found in PCHNBECs, suggesting Z might be more harmful to cancer patients than normal and Z might have adverse health problem when cancer patients intake beef products produced by Z-implanted beef cattle containing Z or its metabolites. One of the mechanisms is due to the increase of DNMT1 and decrease of p53 expression. In addition, all our experimental data was collected from matched human breast tissues, which can provide better data for analyzing the difference between PCHNBECs and PCHBCECs with the same treatment of different concentrations of Z to decrease the possible influence of age, sex, medication, life style, *etc*.

In summary, our data show PCHBCECs may be more sensitive to exposure to Z. The stimulatory effects of Z on PCHBCECs might be possible through down-regulation of the expression of the p53 gene and up-regulation of DNMT1 mRNA and protein. This result suggests that suppressed expression of the p53 gene in PCHBCECs might be mediated through its epigenetic modification. Further investigation into this critical issue is in progress in our laboratory.

## 3. Experimental Section

### 3.1. Isolation of Primary Cultured Human Normal Breast Epithelial Cells (PCHNBECs) and Cancer Epithelial Cells (PCHBCECs)

Human normal and cancerous breast tissues from the same patient were obtained through the Tissue Procurement Program of The Ohio State University Comprehensive Cancer Center Hospital in Columbus, OH, USA. After tissues were transferred in our laboratory, they were minced and then digested using digestion buffer which consisted of phenol red-free high calcium Dulbecco’s modified Eagle’s medium and Ham’s F12 medium (1:1) (DMEM/F12) (1.05 mM CaCl_2_) with 2% Bovine Serum Albumin (BSA) (Invitrogen, Carlsbad, CA, USA) containing 10 ng/mL Cholera toxin (Sigma, St. Louis, MO, USA), 6300 U/mL Collagenase (Invitrogen), and 100 U/mL Hyalurinidase (Calbiochem, Gibbstown, NJ, USA). After the mixture was incubated in a humidified incubator (5% CO_2_, 95% air, 37 °C) overnight, the solution was transferred to a 50 mL tube and centrifuged at 1200 rpm for 5 min. The upper layer containing pre-adipocytes and middle layer containing stromal cells were transferred to another 15 mL tube separately while the pellet containing epithelial cells remained in the tube. All the pellets were washed by DMEM/F12 medium with antibiotic-antimycotic (100 U/mL penicillin G sodium, 100 μg/mL streptomycin sulfate and 0.25 μg/mL amphotericin B) (Invitrogen) and centrifuged again. This wash procedure was repeated three times. The final pellet in the tube contains PCHBCECs or PCHNBECs. The pellet was then resuspended in 10 mL low calcium (0.04 mM CaCl_2_) DMEM/F12 medium supplemented with 10% of low calcium FBS (Atlanta Biologicals, Norcross, GA, USA) and then transferred into a T75 flask for culturing. The method and the specific cultural medium ensure the purity of PCHBCECs or PCHNBECs isolated from normal or cancerous breast tissues. Our lab members have determined the purity of the isolated primary cultured human normal and cancerous breast epithelial cells or stromal cells by using immunocytochemistry [53].

### 3.2. PCHBCECs and PCHNBECs Culture

The isolated PCHNBECs and PCHBCECs were cultured in 75 cm^2^ culture flasks in a humidified incubator (5% CO_2_, 95% air, 37 °C) with 10 mL low calcium (0.04 mM CaCl_2_) DMEM/F12 mixture (Atlanta Biologicals, Norcross, GA, USA) supplemented with 10% of Chelex-100 (Bio-Rad Laboratories, Richmond, CA, USA) treated FBS (Invitrogen). The low calcium DMEM/F12 medium was changed every two days [10]. This was done to ensure the purity of PCHNBECs and PCHBCECs. When the cells grew to 85–90% confluence, cells were washed with 10 mL of calcium- and magnesium-free Phosphate Buffered Saline (PBS, pH 7.3), and then trypsinized with 3 mL of 0.25% trypsin–5.3 mM EDTA (Invitrogen) for 5–8 min at 37 °C. The trypsinization was stopped by addition of 10 mL of DMEM/F12 medium with 10% FBS. After centrifugation, the dissociated cells were resuspended in low calcium DMEM/F12 medium with 10% low calcium FBS and sub-cultured into 75 cm^2^ culture flasks at a ratio of 1 flask to 3 flasks. All experiments were conducted on primary cultured human normal breast epithelial cells not generated beyond the fourth passage.

### 3.3. Cell Proliferation Assay (MTT Assay)

A total volume of 100 μL medium containing 5000 PCHNBECs or PCHBCECs/well was seeded in 96-well plates in low calcium DMEM/F12 medium and incubated at 37 °C for 24 h. The following day, medium was replaced by 100 μL low calcium DMEM/F12 supplemented with 0.2% BSA and incubated at 37 °C for another 24 h. After, the treatment of 7.5, 15, 30 nM Z was given for 24 h (0.1% DMSO to control group), the proliferation of PCHNBECs and PCHBCECs was measured by adding 20 μL of a fresh mixture of 3-(4,5-dimethylthiazol-2-yl)-5-(3-carboxymethoxy-phenyl)-2-(4-sulfophenyl)-2H-tetrazolium (MTS) and phenazine methosulfate (PMS) (20:1) solution (Promega, Madison, WI, USA) to each well. After incubation at 37 °C for 1–3 h, optical density were measured by kinetic microplate reader (Molecular Devices Cooperation, Menio Park, CA, USA) at 490 nm wavelength and cell growth was compared.

### 3.4. Cell Treatment, RNA Isolation and cDNA Synthesis

A total of 1 × 10^5^ viable PCHNBECs and PCHBCECs/well were seeded in 6-well plates in 5 mL low calcium DMEM/F12 medium supplemented with 10% of Chelex-100 (Bio-Rad Laboratories, Hercules, CA, USA) treated FBS (Invitrogen). After 24 h, medium was replaced with low calcium DMEM/F12 supplemented with 10% dextran coated charcoal (DCC) stripped FBS, and the cells were cultured overnight. After PCHNBECs and PCHBCECs were treated with 0.1% DMSO, 7.5, 15 and 30 nM Z for 24 h, total RNA was isolated in 1 mL TRIZOL Reagent (Invitrogen) according to the manufacturer’s instructions. RNA concentration was measured by DU-70 spectrophotometer (Beckman Instruments Inc. Fullerton, CA, USA). RNA (1 μg) from cultured cells was reverse transcribed with 200 UM-MLV Reverse Transcriptase (Invitrogen) at 37 °C for 50 min then 70 °C for 15 min in the presence of 1 μL 10 mM dNTP (10 mM each dATP, dGTP, dCTP, and dTTP at neutral PH) (Invitrogen), 1 μL 50 μM Random hexamer (Amersham, Piscataway, NJ, USA), 10 μL 5× First Strand buffer, 5 μL 0.1 M DTT and 1 μL RNase Inhibitor (Invitrogen) in a total volume of 50 μL in a gradient mastercycle (Eppendorf^®^, Westbury, NY, USA).

### 3.5. Real Time PCR

After cDNA was synthesized, real-time PCR was conducted to amplify p53, DNMT1 and 36B4 genes. The PCR reactions for p53, DNMT1 and 36B4 were performed using the SYBR Green I detection chemistry system and detected with the Stratagene M3005XP system (Agilent Technologies, Cedar Creek, TX, USA). For each PCR run, a mixture contained 10 μL 2× SYBR^®^ Green PCR master mix (Applied Biosystems, Foster City, CA, USA), 2 μL newly synthesized cDNA, 5 μL primer mixer and 3 μL PCR grade water in a total volume of 20 μL. The thermal cycling condition comprised an initial step at 50 °C for 2 min, 95 °C for 10 min, and 45 cycles at 95 °C for 15 s and annealing and elongation at 55 °C for 1 min. Dissociation curves were also conducted after amplification to ensure the reaction specificity. The primer sequences for p53 were 5′-GCT CCT GTG CTG CGA AGT GG-3′ (sense) and 5′-TGG AGG CGT CGG TGT AGA TG-3′ (antisense, product size 372 bp). The primer sequences for DNMT1 were 5′-CAT TTT ATC CCC ATT GAG AAG TA-3′ (sense) and 5′-CTG AAA ATT AAG TCC TTG TGC CCA G-3′ (antisense, product size 273 bp). The primer sequences for 36B4, an internal control were 5′-AAA CTG CTG CCT CAT ATC CG-3′ (sense) and 5′-TTT CAG CAA GTG GGA AGG TG-3′ (antisense, product size 563 bp). The results were presented as the ratio of p53 or DNMT1 to 36 B4.

### 3.6. Western Blotting Assay

PCHBCECs and PCHNBECs were plated in a 10 cm culture dish with a density of 1 × 10^6^ viable cells/dish within 10 mL low calcium DMEM/F12 supplement with 10% FBS and cultured overnight. The media was replaced with low calcium DMEM/F12 supplemented with 5% DCC treated FBS and was cultured for another 24 h. PCHBCECs and PCHNBECs treatment were the same as previously described. After 24 h treatment, proteins were extracted from each treatment group using M-PER^®^ mammalian protein extraction reagent (Pierce, Rockford, IL, USA) according to the manufacturer’s instructions. Protein concentrations were measured using a Micro BCA™ protein assay reagent kit (Pierce, Rockford, IL, USA) following the manufacturer’s protocol. Proteins (50 μg) from each treatment group were separated by 4–15% Tris-HCl gel electrophoresis and then transferred to a Polyvinylidine Fluoride (PVDF) membrane (Bio-Rad Laboratory, Hercules, CA, USA). The membrane was first blocked in Phosphate Buffered Saline −0.1% Tween 20 (PBST) containing 10% fat free dry milk for 1 h and then incubated with DNMT1 goat polyclonal antibody (dilution 1:500, sc-10219, Santa Cruz Biotechnology, Inc. Santa Cruz, CA, USA), p53 antibody (1:1000 dilution, Cell Signaling Technology^®^ Danvers, MA, USA) and β-actin antibody (1:2000 dilution, Santa Cruz Biotechnology, Inc.) for 1 h. The membrane was rinsed in PBST three times, each time for 5 min. In the following step, the membrane was incubated with second antibody (donkey anti-goat IgG-horseradish peroxidase HRP for DNMT1 and β actin; ECLTM anti-rabbit IgG-HRP for detecting p53) for 1 h. After washing the membrane in PBST three times, p53, DNMT1, and β-actin protein were visualized with a chemiluminescent detection system (ECL, Amersham Pharmacia Biotech, Buckinghamshire, UK). Pictures were taken using a FujiFilm LAS-300 imaging system (FUJIFILM Medical Systems USA, Inc.). The protein ratios of p53 to β-actin and DNMT1 to β-actin were calculated by measuring the density of the specific band using Multi-Gauge software (v3.0).

### 3.7. Statistical Analysis

The results for the cell proliferation assay are presented compared to the control group as mean ± standard deviation (SD) for 4 replicate culture wells. The results for the mRNA expression are presented compared to the control group as mean ± standard deviation (SD) for 3 replicates. Analyses were performed using Minitab 15 software (Minitab Inc. PA, USA). Statistical differences were determined by using two sample *t*-test or ANOVA analysis for independent samples. *P*-values of less than 0.05 were considered statistically significant differences.

## 4. Conclusions

Our experimental results demonstrated that the growth promoter Z stimulates the proliferation of PCHBCECs. The stimulatory effects of Z on the cells may be mediated by down-regulating the tumor suppressor gene p53 at the mRNA and protein levels. Further investigation illustrated that the DNA methylatransferase 1 mRNA and protein levels were up-regulated by the treatment of Z in PCHBCECs as compared to PCHNBECs, which suggests a role of Z in epigenetic modification involved in the regulation of p53 gene expression in PCHBCECs. Our experimental results imply the potential adverse health effect of Z in breast cancer development. Further study is under process in our laboratory
